# The architecture of amyloid-like peptide fibrils revealed by X-ray scattering, diffraction and electron microscopy

**DOI:** 10.1107/S1399004715001674

**Published:** 2015-03-27

**Authors:** Annette E. Langkilde, Kyle L. Morris, Louise C. Serpell, Dmitri I. Svergun, Bente Vestergaard

**Affiliations:** a Department of Drug Design and Pharmacology, University of Copenhagen, Universitetsparken 2, DK-2100 Copenhagen, Denmark; b School of Life Sciences, University of Sussex, Falmer, Brighton, England; c European Molecular Biology Laboratory, Hamburg Outstation, 22607 Hamburg, Germany

**Keywords:** amyloid-like fibril, fibril structure, small-angle X-ray scattering, fibre diffraction, electron microscopy, hybrid structural analysis, hierarchical assembly

## Abstract

Structural analysis of protein fibrillation is inherently challenging. Given the crucial role of fibrils in amyloid diseases, method advancement is urgently needed. A hybrid modelling approach is presented enabling detailed analysis of a highly ordered and hierarchically organized fibril of the GNNQQNY peptide fragment of a yeast prion protein. Data from small-angle X-ray solution scattering, fibre diffraction and electron microscopy are combined with existing high-resolution X-ray crystallographic structures to investigate the fibrillation process and the hierarchical fibril structure of the peptide fragment. The elongation of these fibrils proceeds without the accumulation of any detectable amount of intermediate oligomeric species, as is otherwise reported for, for example, glucagon, insulin and α-synuclein. Ribbons constituted of linearly arranged protofilaments are formed. An additional hierarchical layer is generated *via* the pairing of ribbons during fibril maturation. Based on the complementary data, a quasi-atomic resolution model of the protofilament peptide arrangement is suggested. The peptide structure appears in a β-sheet arrangement reminiscent of the β-zipper structures evident from high-resolution crystal structures, with specific differences in the relative peptide orientation. The complexity of protein fibrillation and structure emphasizes the need to use multiple complementary methods.

## Abstract 2

The aggregation process and the fibril state of an amyloidogenic peptide suggest monomer addition to be the prevailing mechanism of elongation and a model of the peptide packing in the fibrils has been obtained.

## 1. Introduction   

The severity of several amyloid diseases underlines the importance of studying the structural aspects of protein amyloid fibrillation (Cecchi & Stefani, 2013 ▶; Knowles *et al.*, 2014 ▶). However, in spite of more than a century of dedicated research efforts, how amyloid-like fibrils are formed remains elusive. This is mainly because fibrillation constitutes an inherent structural analytical challenge, since fibril formation proceeds *via* several equilibria between native and unfolded or refolded structures, oligomers, protofilaments and mature fibrils. Protein fibrils are however not only relevant in the context of diseases, as in addition these complex self-assembly nanostructures are promising scaffolds for the future development of biocompatible nanomaterials with an expected wide range of applications (Gras, 2007 ▶).

Many fragments of amyloidogenic proteins as well as synthetic peptides can form amyloid-like fibrils *in vitro*. Compared with fibrils formed from full-length amyloidogenic proteins, such peptide fibrils exhibit relatively lower complexity and hence provide ideal model systems for structural analysis (see, for example, Balbirnie *et al.*, 2001 ▶; Reches *et al.*, 2002 ▶).

The identification of particular sequences defining fragments with high amyloidogenic propensity aids in identifying core regions which are potentially essential for the fibrillation of the full-length proteins. It is thus expected that structural information from fibrils of such shorter fragments will reflect aspects of the corresponding full-length protein fibrils, and hence may be of importance to the understanding of the mechanism behind fibrillation of the corresponding various disease-relevant proteins. The advantages of using shorter peptide fragments are immediately obvious, since the shorter and simplified systems offer the study of a fibril with reduced complexity yet reflecting the major important aspects of the full-length complex fibrils. Here, an amyloidogenic fragment of a yeast prion protein, GNNQQNY, is predicted to form the fibril core in prion fibrils. Our study may thus ultimately improve the understanding of the related prion diseases. Yeast prion proteins, both as fragments and the full-length protein, have been used as models for prion systems as well for general amyloid diseases (Wickner *et al.*, 2013 ▶). The native protein product of Sup35p takes part in the termination of translation (Wickner *et al.*, 2013 ▶), but has an N-terminal prion domain rich in asparagine and glutamine residues. The heptapeptide GNNQQNY corresponds to a fragment of this N-terminal domain (Sup35p_7–13_), and the importance of the GNNQQNY fragment in the fibrillation of full-length Sup35p has been indicated by cross-seeding experiments (Balbirnie *et al.*, 2001 ▶). Several studies have reported a concentration-dependent polymorphism of the possible solution states of this peptide, revealing the formation of either fibrils or nano­crystals (Balbirnie *et al.*, 2001 ▶; Diaz-Avalos *et al.*, 2003 ▶). The formation of nanocrystals facilitated the determination of the atomic structures of a number of amyloidogenic peptides, including GNNQQNY (Nelson *et al.*, 2005 ▶; Sawaya *et al.*, 2007 ▶; Wiltzius *et al.*, 2008 ▶), revealing that peptides in the crystals are arranged in a so-called steric zipper or dry zipper (β-sheets with tight packing of side chains). It is suggested that the structural motifs observed in these crystals are closely related to the core structure of the fibrils. Hence, some of these peptides, such as GNNQQNY, have become model systems contributing to an understanding of the mechanism and driving forces in protein fibril formation. Extensive studies of the GNNQQNY peptide, including crystal structures, solid-state nuclear magnetic resonance spectroscopy (ss-NMR) of both crystal and fibril forms, computational studies and a wide range of biophysical characterization, have all elucidated different structural aspects of the peptide (Nelson *et al.*, 2005 ▶; van der Wel *et al.*, 2007 ▶; Debelouchina *et al.*, 2010 ▶; Nasica-Labouze *et al.*, 2011 ▶; Marshall *et al.*, 2010 ▶; Qi *et al.*, 2012 ▶). However, there are distinct structural differences between fibrils and crystals (Marshall *et al.*, 2010 ▶; van der Wel *et al.*, 2007 ▶). The relevance of the crystal structure has been investigated using molecular-dynamics (MD) simulations (Periole *et al.*, 2009 ▶; Esposito *et al.*, 2006 ▶), which indicated that the crystal β-zippers can twist into fibril-like structures *via* only minor rotations between the β-strands. Other experimental data have shown a fibril-to-crystal transformation and have revealed significant differences in the diffraction from the two forms that was not explicable by twisting of the β-sheets alone but also by the environment of the tyrosine residue (Marshall *et al.*, 2010 ▶). These partially conflicting studies may reflect that fibrillation may lead to more than one specific fibril structure, and hence that the crystal structures are likely to be closely related to some, but not all, such fibrillar forms. Indeed, MD studies show that some peptides, and GNNQQNY in particular, form stable structures in several zipper arrangements (Berryman *et al.*, 2009 ▶, 2011 ▶). Likewise, it has been observed using magic angle (MAS) NMR and ss-NMR that the fibrils exhibit a structural complexity beyond that of the crystals (van der Wel *et al.*, 2007 ▶, 2010 ▶; Lewandowski *et al.*, 2011 ▶). The actual peptide packing in the fibril form is thus still enigmatic.

A recent publication demonstrated a tour de force in hybrid structural analysis and provided atomic resolution details from the fibril arrangement of a transthyretin-derived 11-residue peptide (Fitzpatrick *et al.*, 2013 ▶). This demonstrates the full potential of combining different methods spanning several orders of magnitude in the structural analysis of amyloid-like fibrils. This impressive analysis method, including extensive MAS NMR analyses, is however extremely demanding both in material and time. In the present study, we use an alternative hybrid approach to investigate the structural properties of GNNQQNY fibrils. In our approach, by applying advanced solution small-angle X-ray scattering (SAXS) as the central method, we enable detailed interpretation of high-quality fibre diffraction (FD) in a more time-efficient and material-efficient manner. Transmission electron microscopy (TEM) experiments support and validate the analysis, and the SAXS data collected successively at numerous time points throughout the fibrillation process furthermore provide insight into the structural maturation of fibrils. This method also investigates any potential presence of additional transiently formed oligomeric species (Vestergaard *et al.*, 2007 ▶; Oliveira *et al.*, 2009 ▶; Giehm *et al.*, 2011 ▶; Langkilde & Vestergaard, 2012 ▶). Our data show that the peptide forms highly ordered laminar macroscopic structures, and suggest that aggregation proceeds without significant accumulation of transient peptide oligomers. Most importantly, supported by the available high-resolution crystal structures, the analysis enables the quasi-atomic resolution modelling of the hierarchically formed GNNQQNY peptide fibril.

## 2. Materials and methods   

### 2.1. Peptide   

The heptapeptide GNNQQNY was purchased from Caslo A/S as the trifluoroacetate salt with a purity of >98%.

### 2.2. Fibrillation assay   

Two different protocols were applied. The peptide was dissolved in H_2_O to final concentrations of 8.7 and 8.6 mg ml^−1^ and filtered through a 22 µm filter. Alternatively, the peptide was dissolved in DMSO and diluted with water to 10%(*v*/*v*) DMSO to give final peptide concentrations of 5.8 and 6.1 mg ml^−1^ and filtered. At such peptide concentrations the lag phase is significantly shortened; hence, cold water (4°C) was used for dilution to delay the initial processes. Peptide concentrations were determined based on *A*_280_ using ∊ = 1280 cm^−1^ *M* ^−1^. Thioflavin T (ThT) was added to a final concentration of 40 µ*M*. The solutions were incubated in 96-well plates (Nunc) at 32°C (samples in H_2_O) or 35°C (samples in 10% DMSO) in a BMG PolarStar Fluorescence Plate reader following fluorescence emission at 480 ± 5 nm upon excitation at 450 ± 5 nm. It was hypothesized previously that supercritical concentrations are required for the accumulation of structural nuclei (Powers & Powers, 2006 ▶). In this study the supercritical concentration could not be determined, yet peptide concentrations were used which caused an almost complete bypass of the lag phase.

### 2.3. Sonication   

The peptide samples became very viscous at later time points in the fibrillation process corresponding to increased amounts of fibrillar material. Hence, sonication was necessary in order to obtain scattering data from a solution of randomly oriented fibrils (Langkilde & Vestergaard, 2012 ▶). Here, 10 s of pulsed sonication was applied using a Sonopuls 2270 (Bandelin).

### 2.4. Small-angle X-ray scattering (SAXS) data collection   

The fibrillation process was started in a large batch of adequate volume to allow analysis at several time points. The content of the wells was extracted at different time points from the plate reader as the fibrillation process was followed by fluorescence spectroscopy, extracting one sample per well for immediate subsequent SAXS data collection. The details of this approach have been described elsewhere (Langkilde & Vestergaard, 2012 ▶). Additional late data points from a fourth fibrillation series (also in 10% DMSO) were included in parts of the analyses (10.7 and 13.1 h). All SAXS data were collected on the EMBL SAXS beamline X33 at the DORIS storage ring, DESY, Hamburg, Germany (Blanchet *et al.*, 2012 ▶; Franke *et al.*, 2012 ▶) within an *s* range of 0.08–5 nm^−1^ using a PILATUS 1M detector. The momentum transfer *s* is given by *s* = 4π(sinθ/λ), where 2θ is the scattering angle and the wavelength λ is 0.15 nm. The sample-to-detector distance was 2.7 m and measurements were performed with 120 s exposures. The corresponding real-space distances probed are *d* ∈ (90 nm, 1.2 nm).

### 2.5. SAXS data-evaluation procedures   

Two-dimensional images of the fibrillation measurements were visually inspected using *FIT*2*D* (Hammersley, 1997 ▶) and the corresponding data were discarded before further analysis if the two-dimensional images revealed non-isotropic scattering. Data that were not discarded were corrected for detector response and scaled according to protein concentration, exposure time and intensity before radial averaging. Repeated exposures (4 × 30 s) revealed no sign of X-ray-induced aggregation, with the exception of the two latest time points, where only the first two exposures were used in the subsequent analysis. After buffer subtraction, Guinier analysis was performed using *PRIMUS* (Konarev *et al.*, 2003 ▶), and the first usable data point from this analysis was used as *s*_min_ for the given measurement. For the significantly fibrillated samples, a Guinier range as defined by *s*_min_ *R*_g_ < 1.3 was not obtained, and only a rough estimate could be made. Indirect Fourier transformation was then performed in *GNOM* (Svergun, 1992 ▶) using an *s*_max_ of 4 nm^−1^ (for the globular approach). The fit of the monomeric peptide to the data from the starting conditions was tested using *CRYSOL* (Svergun *et al.*, 1995 ▶) with GNNQQNY monomers from PDB entry 2omm, as well as dimers created from the structure as a pair from neighbouring β-sheets and a pair within the same β-sheet to test the level of distinction. The position of the Bragg peak and the corresponding Bragg spacing were evaluated using *PEAK* (Konarev *et al.*, 2003 ▶).

### 2.6. Multi-component analysis of SAXS data   

Singular value decomposition (SVD) analysis was performed using the routine included in *PRIMUS* (Konarev *et al.*, 2003 ▶). Data with *s* > 0.3 nm^−1^ from fibrillation series with and without 10% DMSO were included in two different runs with and without the two latest time points. Fitting using linear combinations of start and end points was performed using *OLIGOMER* (also included in *PRIMUS*; Konarev *et al.*, 2003 ▶). The oligomer analysis was performed on the pool of data using the theoretical scattering from the monomeric peptide (as obtained from the *CRYSOL* fit mentioned above) together with the 9.0 h sample (a late stage in the fibrillation series, with the final level of ThT, and thus expected to be fully fibrillated) as input components. In addition, a three-component oligomer analysis was additionally performed using the 10.7 h sample as the third input component.

### 2.7. Fitting geometrical shapes to the SAXS data   

Using *BODIES* available within *PRIMUS* (Konarev *et al.*, 2003 ▶), simple geometrical shapes can be fitted against the individual scattering curves. This was performed for all data collected from samples extracted more than 1 h after initiation of the fibrillation process. Only data with *s* < 1.5 nm^−1^ were used in this analysis.

### 2.8. Cross-section analysis and mass per unit length based on SAXS data   

For very elongated particles, the scattering contribution of the long axis (the fibril axis in this case) can be separated from that of the cross-section (Feigin & Svergun, 1987 ▶) and the cross-section of such species can be evaluated individually using approaches similar to those regularly used for globular species. Guinier analysis was first performed using *PRIMUS* (Konarev *et al.*, 2003 ▶). From the intercept at *s* = 0 in the Guinier plot for a rod-like particle (Supplementary Figs. S1*a*, S1*b* and S1*c*), lim_s→0_[*sI*(*s*)] is determined. The mass per unit length (*M*_L_) can be calculated using the forward scattering [*I*(0)] of a standard protein sample, in this case bovine serum albumin (BSA): *M*_L_ = {lim_s→0_[*sI*(*s*)]MW_BSA_}/[*I*(0)_BSA_π]. In this calculation, the partial specific volumes of the sample and the standard are assumed to be identical. The uncertainty in *M*_L_ is not only dependent on the SAXS data quality [*I*(0) determination of both sample and BSA] but also on the concentration determination of both the sample and BSA. The actual error in such a mass estimate can therefore vary from a few percent upwards, and in this case it is not reasonable to make an explicit statement. Additionally, indirect Fourier transformation was then performed using *GNOM* (Svergun, 1992 ▶) with an *s*_max_ of 1.5 nm^−1^ and a rod-like assumption (a built-in option in *GNOM*), thereby evaluating the pair-distance distribution of the cross-section alone.

### 2.9. Transmission electron microscopy (TEM)   

Samples of untreated and sonicated fibrils of GNNQQNY (from fibrillation in 10% DMSO with 40 µ*M* ThT) were examined. 5 µl sample was allowed to adsorb onto a Formvar/carbon 300 Mesh Cu Grid (Agar Scientific) for 1 min before blotting and washing with with 5 µl water in a 1 min incubation. Adsorbed material was negatively stained by two 1 min incubations with 5 µl 2% uranyl acetate. TEM was performed using a H-7100 transmission electron microscope (Hitachi) and images were acquired digitally using an axially mounted UltraScan 1000 CCD camera (Gatan). Ribbon widths were measured using the *GNU Image Manipulation Program* and an average striation width was determined.

### 2.10. Fibre diffraction (FD)   

Dry aligned samples were obtained by placing a drop of 5–10 µl fibrillated peptide solution between two closed capillaries with their ends a few millimetres apart to simulate the stretch-frame approach to align the sample (Morris & Serpell, 2012 ▶). The droplet was left to dry overnight. The dried fibril samples were then mounted on a standard macromolecular crystallography pin. Fibre diffraction data were collected on MAX-lab beamlines I911-3 (λ = 0.099 nm, sample-to-detector distance 290 mm, MAR 225 detector) and I911-2 (λ = 0.104 nm, sample-to-detector distance 220 mm, MAR 165 detector). All data were collected at 4°C with exposure times of 30–120 s per frame in both static mode and with 90° rotation around the fibril long axis during exposure. Equatorial and meridional signals were plotted by radially integrating 60° of data about each respective axis using the radial average function of *CLEARER* (Makin *et al.*, 2007 ▶). Integrated diffraction signals were exported as a function of pixels and converted to real-space distances *d* using Bragg’s law.

### 2.11. Indexing of FD patterns   

X-ray fibre diffraction reflections were measured using *CLEARER* (Makin *et al.*, 2007 ▶) and were combined with equatorial data from previously reported patterns of GNNQQNY (Marshall *et al.*, 2010 ▶). Possible unit-cell dimensions were explored using *CLEARER* (Makin *et al.*, 2007 ▶). The observed Bragg peak of 4.8 nm from the SAXS data was also included as an equatorial signal to obtain indexing (Supplementary Table S1).

### 2.12. Crystallization and diffraction   

The sample was prepared as for the fibrillation assay with DMSO present and was left at room temperature in a 1.5 ml Eppendorf tube, in which several bundles of needle-like crystals were formed. A bundle of these needle-shaped crystals was mounted on a pin like the dry fibril samples and diffraction data were collected as described above.

### 2.13. Simulation of diffraction patterns   

*CLEARER* (Makin *et al.*, 2007 ▶) was also used to simulate diffraction patterns from crystal structures (PDB entries 2omm and 1yjp; Sawaya *et al.*, 2007 ▶; Nelson *et al.*, 2005 ▶) and the model of the peptide packing using the indexed unit cell. Settings for the simulation were set to match the experimental setup. For simulations based on the fibre models, the crystallite size was set to mimic the ribbon size determined (approximately 40 × 200 × 6 nm).

### 2.14. Modelling peptide packing   

Starting from the crystal structure conformation of PDB entry 2omm (Sawaya *et al.*, 2007 ▶), the side-chain configurations of Asn6 and Tyr7 were changed using the Dunbrack rotamer library (Dunbrack, 2002 ▶) to facilitate parallel packing of the sheets. A parallel pair of peptides was built roughly based on the backbone positions of PDB entry 2omm (Sawaya *et al.*, 2007 ▶) and a copy of this pair rotated to model the suggested packing. Optimization and addition of water molecules was performed using *AMBERtools* in *UCSF Chimera* (Pettersen *et al.*, 2004 ▶). Water molecules outside the unit-cell boundary were subsequently deleted to obtain the model used for the simulation of FD. A ribbon model was constructed from the basis of this unit cell using *PyMOL* (Schrödinger).

## 3. Results   

### 3.1. No structural nucleus is observed during the fibrillation process   

A monomeric starting point is revealed by the SAXS data immediately after dissolution and filtration of the sample. The estimated molecular weight (MW) and radius of gyration (*R*_g_) are in agreement with those of a monomeric peptide, and the theoretical scattering curve calculated from the monomeric peptide (from PDB entry 2omm; Sawaya *et al.*, 2007 ▶) fits the experimental data (Supplementary Fig. S2).

**Figure 1:**
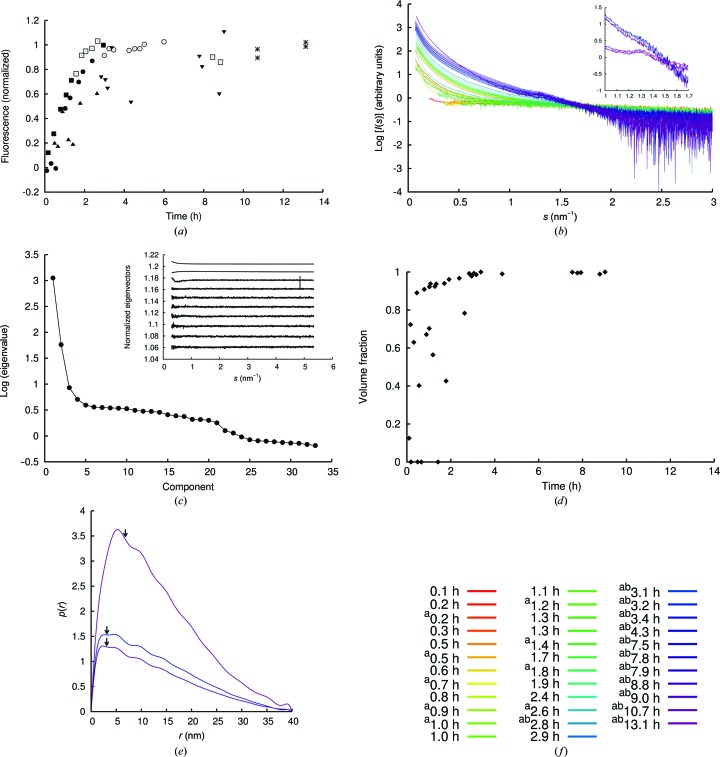
Data from the fibrillation process of GNNQQNY. (*a*) Normalized ThT fluorescence emission intensity recorded at the time that the samples were extracted from the fluorescence plate reader. Three fibrillation series are included: 8.7 and 8.6 mg ml^−1^ peptide in H_2_O (squares and circles) and 5.8 mg ml^−1^ peptide in 10% DMSO (triangles). Closed symbols correspond to the SAXS data used in the following analysis, while open symbols represent non-isotropic (and hence discarded) scattering data. Samples sonicated prior to SAXS data collection are shown by inverted triangles. Additionally, late measurements (stars; 10.7 and 13.1 h) from fibrillation of 6.1 mg ml^−1^ peptide in 10% DMSO were measured. SAXS data were obtained from samples pooled from two wells, and these data are only partially included in the following analysis (see §[Sec sec3]3 for details). (*b*) SAXS data from the extracted samples [corresponding to filled symbols and stars in (*a*)]. Inset: enlargement of the data from 3.4 and 9.0 h showing increased intensity around *s* = 1.3 nm^−1^, while the data from the 10.7 and 13.1 h samples show a clear Bragg peak at *s* = 1.3 nm^−1^. (*c*) Eigenvalues from singular value decomposition (SVD), excluding the two late time points (10.7 and 13.1 h). Inset: the first ten eigenvectors from the SVD analysis. (*d*) Fibril volume fractions obtained from *OLIGOMER* analysis using the theoretical monomer and the 9.0 h samples as representatives of the two components. (*e*) Cross-sectional pair-distance distribution functions for the samples at 3.4, 9.0 and 10.7 h. (*f*) The colour scale from red to purple used in (*b*) and (*e*) to show the development over time. A superscript ‘a’ indicates that the fibrillation conditions included 10% DMSO and a superscript ‘b’ indicates that the sample was sonicated immediately before measuring the SAXS data.

The fibrillation process was followed using thioflavin T (ThT) fluorescence (Fig. 1 ▶ *a*). In H_2_O the fibrillation process initiated immediately; thus no ThT baseline could be measured and the SAXS data measured as soon as possible after dissolution and filtration included non-monomeric signal. When fibrillating the peptide in 10% DMSO, it was possible to obtain ThT and SAXS data from the lag phase (*i.e.* before fibril formation and the elongation phase). In the samples with DMSO present, a larger variation in initiation of the elongation phase was observed (triangles in Fig. 1 ▶ *a*); however, all samples followed a similar steep elongation phase after onset. The fibrils obtained using these two slightly different conditions were compared on overall morphology as well as internal structure (discussed below). SAXS data were measured at different time points in the fibrillation process. The progressive increase in scattering intensity at low angles is clear proof of the development of very large species (Fig. 1 ▶ *b*). The nature of the scattering curve changes from almost flat to convex curves correlated with a clear change in the nature of the scattering species in solution. Notably, for the samples reaching the ThT plateau increased scattering around *s* = 1.3 nm^−1^ is observed, and at the very late time points a distinct Bragg peak at this position is evident from the scattering profiles (Fig. 1 ▶ *b*). A peak at this position corresponds to a real-space distance of 4.8 nm, revealing the presence of a highly repetitive distance within the developing fibrils. Likewise, estimates of *R*_g_ and the maximum dimension of the scatterer (*D*_max_) at all time points (Supplementary Fig. S3*b*) reveal the development of the average and maximal sizes of the scattering particles that are present in solution. *D*_max_ is obtained during the indirect Fourier transformation to the pair-distance distributions (Supplementary Fig. S3*c*) but, like *R*_g_, can only be estimated with some uncertainty when the length of the fibrils surpasses the resolution of the SAXS data.

The scattering contributions from different (non-interacting) species in the sample are additive; thus, applying singular value decomposition (SVD) to the accumulated data from the process can reveal the number of species present, and the scattering contribution from each individual species can be isolated by careful data analysis (Langkilde & Vestergaard, 2012 ▶). This approach has previously been used to describe structural nuclei in the fibrillation of insulin (Vestergaard *et al.*, 2007 ▶), glucagon (Oliveira *et al.*, 2009 ▶) and α-synuclein (Giehm *et al.*, 2011 ▶). SVD of the collected pool of GNNQQNY SAXS data (excluding the two latest time points) shows the presence of two dominating and a minor third scattering species (Fig. 1*c* ▶). If the solution contains two major components (here monomers and fibrils), the scattering at different time points will be a linear combination (scaled by the volume fractions) of the scattering curves representing the two individual components. To test this hypothesis, scattering curves at the start and end points are required. The sample representing the earliest time point (the sample in DMSO at 0.2 h) showed a monomeric character (Supplementary Fig. S2) and the theoretical curve fitted to this starting point was used as the first component. A late sample (9.0 h), corresponding to the ThT plateau, was selected as the end point (representing the fibril sample). As is seen, data from the intermediate time points can be consistently fitted as a linear combination of these two components, resulting in very limited and nonsystematic residual scattering at the latest time points (Supplementary Fig. S4). From this analysis, the corresponding volume fractions of the two components are obtained (Fig. 1*d* ▶ and Supplementary Table S2) and it can be seen that samples collected after 3.0 h are predominantly fibrils. This also supports the assumption that the 9.0 h sample is a proper representatation of the fibrillar state. We thus have no indications of the presence of additional species during the elongation phase and we conclude that the indication of a third minor species from the SVD is negligible at all time points until 9.0 h. However, when including the two latest time points (10.7 and 13.1 h) a third species is more prominent (Supplementary Figs. S5 and S6). The most natural explanation of this result is that a third species which is not present in significant amounts during the elongation of fibrils accumulates during the maturation phase.

Based on this analysis, we conclude that no detectable amount of intermediate oligomers is present during this fibrillation process. The elongation process is thus most likely to proceed *via* monomer addition.

### 3.2. The ribbon architecture of GNNQNNY fibrils   

**Figure 2:**
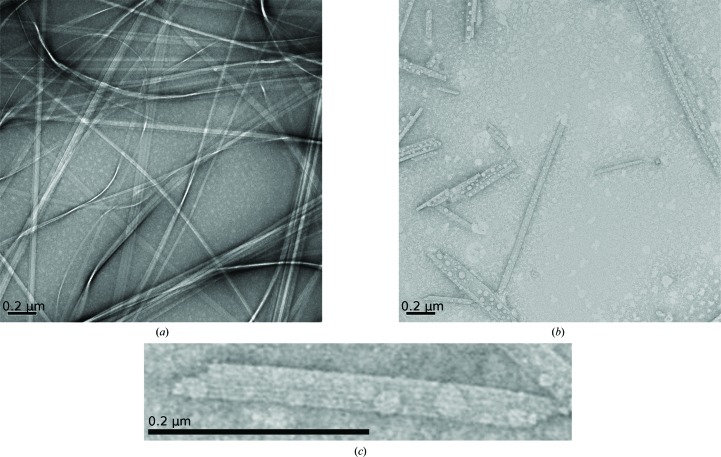

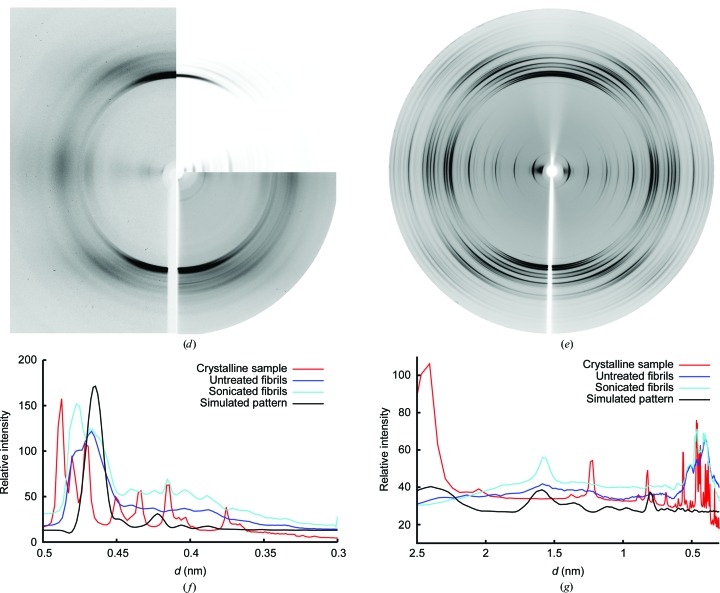
TEM images and diffraction patterns. (*a*) TEM of untreated fibrils and (*b*) sonicated fibrils, as well as (*c*) an enlargement from the sonicated sample showing the lateral striations and exposed ends of the individual filaments in the ribbon. The scale bars in (*a*)–(*c*) are all 0.2 µm. (*d*) Corresponding fibre diffraction patterns from dried samples of untreated fibril sample (left half) and sonicated fibrils (lower right quadrant). The simulated diffraction pattern based on the determined unit cell and the suggested packing model is included (upper right quadrant). (*e*) The diffraction pattern from a bundle of partly aligned needle-shaped crystals. Radial averages of the experimental diffraction images and the simulated pattern in the meridional (*f*) and equatorial (*g*) sections. For the meridional direction, only a selected range is depicted as the patterns are essentially featureless until 0.5 nm.

In previous SAXS studies of fibrils, the fibril macroscopic structure has been modelled as beads on a string, where a macroscopic repeating unit (Vestergaard *et al.*, 2007 ▶; Giehm *et al.*, 2011 ▶; Langkilde & Vestergaard, 2009 ▶) directly related to the overall pitch of twisting of fibrils enables modelling of the entire fibril. In the present case, such a pitch appears to be either larger than the detectable range or alternatively there is no well defined repeat. Indeed, TEM images also only reveal limited and irregular twisting of the ribbons (Fig. 2 ▶ *a*). Kratky plots (Supplementary Fig. S3*d*) of the data reveal well defined structural information below 90 nm.

When fitting the data with a simple geometrical shape using *BODIES* in *PRIMUS* (Konarev *et al.*, 2003 ▶), the best approximations are obtained with either a cylindrical ellipsoid or a parallelepiped (Supplementary Figs. S7*a* and S7*b* and Supplementary Table S3). For the samples extracted later than 3 h both shapes are dominated by one short cross-sectional dimension, a longer second axis (roughly 35–40 nm and corresponding to twice the elliptical *b* semi-axis or the parallelepiped *b* axis) and a long third axis (approximately 55 nm and corresponding to the direction of the fibril long axis; given as the *c* axis in Supplementary Table S3). Interestingly, still focusing on the samples extracted later than 3 h, the dimensions of these shapes vary almost only in one dimension, namely the shortest axis (*a* axis; Supplementary Table S3), which is close to either 5 or 9 nm. Again, the longest axis in particular can only be determined with uncertainty, and is not applicable in the continued analysis. This approach using geometrical shapes to describe the scattering species is a very simplified, and the dimensions obtained do vary (Supplementary Table S3), but the approach gives indications about the overall dimensions and morphology of these samples.

Together with the shape of the scattering curve, this initial analysis underlines the elongated nature of the mature fibrils. Importantly, for very elongated particles the scattering contribution of the long axis (the fibril axis in this case) can be separated from that of the cross-section (Feigin & Svergun, 1987 ▶); hence, the cross-section can be analyzed in detail even if the longest axis of the scatterer is not resolved. The corresponding cross-section *P*(*r*) function (Fig. 1 ▶ *e*) reveals an elongated and slim shape with a maximum dimension of approximately 40 nm, in accordance with the simple geo­metrical analysis. Performing this analysis for samples from different time points results in pair-distance distributions with a maximum shifted to the left (Fig. 1 ▶ *e*). This indicates an elongated cross-section with dimensions of approximately 3 × 40 nm (samples from 3–9 h) to 6 × 40 nm (for the two latest samples). The dimensions are deduced from the plot as the inflection point immediately after the first maximum (Feigin & Svergun, 1987 ▶; arrows in Fig. 1 ▶ *e*) and the maximal dimension. Explicit error estimation on these distances is not possible, but the identification of the inflection point is complicated in this case by additional ripples on the distributions, and the maximum dimension observed may depend on the resolution of the data. The ripples observed in the cross-sectional *P*(*r*) functions (Fig. 1 ▶ *e*) are separated by approximately 5 nm. The spacing between these repeating distances thus corresponds to the Bragg peak in the raw data (Fig. 1 ▶ *b*; highlighted in the inset) and it is concluded that the cross-section is assembled from building blocks with this diameter. Building blocks that are repeated across the cross-section must correspond to individual protofilaments, hence the width of these is 4.8 nm. We thus conclude that the fibril structure has a ribbon-like appearance with a lateral assembly of protofilaments.

TEM images clearly confirm the presence of a ribbon-like macroscopic structure (Figs. 2 ▶ *a*, 2 ▶ *b* and 2 ▶ *c*). Also, clear striations with a width of 5.04 ± 0.26 nm (Supplementary Fig. S8 and Supplementary Table S4) are observed perpendicular to the longest axis of the ribbons. This corresponds to the expected protofilament width of 4.8 nm (as determined from the SAXS data). Upon close inspection of the exposed ends of ribbons (Fig. 2 ▶ *c*) from sonicated samples, the notion of individual protofilaments arranged side by side in the ribbons is further substantiated. It is also evident from the TEM images that there is some variation in the number of protofilaments in each ribbon. In conclusion, the ribbons are composed of a linearly organized set of individual protofilaments.

### 3.3. Probing the internal order of fibrils and crystals   

Firstly, X-ray fibre diffraction (FD) was applied to partially aligned untreated and sonicated GNNQQNY fibrils (Fig. 2 ▶ *d*), thereby allowing a decisive analysis as to whether sonication had an effect on the internal structure of individual protofilaments. The patterns showed the expected cross-β peaks and a close match in the peak positions (Figs. 2 ▶ *f* and 2 ▶ *g*). The TEM analysis of sonicated and nonsonicated samples showed that the length of the striated ribbon is clearly influenced by sonication, while the laminar assembly appears to remain intact. The FD analysis covers a resolution range of approximately 3–0.3 nm and thus conclusively shows that the internal structure of the protofilaments is also conserved upon sonication, thus also validating the SAXS analysis from sonicated fibril samples.

Two polymorphic crystal structures of GNNQQNY have previously been determined (Nelson *et al.*, 2005 ▶; Sawaya *et al.*, 2007 ▶). During this study, needle-like crystals were also obtained (see §[Sec sec2]2); however, the pattern collected from a bundle of these aligned needle-shaped crystals (Fig. 2 ▶ *e*) did not match simulated diffraction data based on the deposited structures (Supplementary Figure S9), implying the existence of further polymorphic crystal forms. At no point did we observe a conversion from the crystal to the fibril form or *vice versa*. The crystals and fibrils observed in this study were stable for months.

Although the crystals show a cross-β-like pattern, a higher degree of order is evident from the larger number of well defined peaks. Comparing the diffraction patterns (Figs. 2 ▶ *f* and 2 ▶ *g*) it is clear that the fibrils and the crystals differ, and as also reported in a previous study (Marshall *et al.*, 2010 ▶) the GNNQQNY fibril FD data do not match simulated patterns based on the deposited crystal structures (PDB entries 2omm and 1yjp; Sawaya *et al.*, 2007 ▶; Nelson *et al.*, 2005 ▶). The crystal forms of this peptide thus cannot directly model the fibril packing, but may serve as the basis for modelling a possible packing scheme.

### 3.4. Determination of the basic unit of the fibrils   

With the aim of determining the quasi-atomic resolution fibril structure, a detailed analysis of the FD data from GNNQQNY fibrils was undertaken. We observed a cross-β signature of amyloid fibrils (Fig. 2 ▶ *d*) with a 0.47 nm distance between consecutive β-strands in the continuous β-sheets along the fibril axis. The equatorial FD data of sonicated fibrils reveal signals at 1.60, 1.35, 0.928, 0.808, 0.754, 0.699, 0.603, 0.511, 0.464 and 0.404 nm; however, it is not possible to unequivocally index the data, *i.e.* the unit cell cannot be assigned unambiguously, in part because the fibril cross-section information is in the rather diffuse equatorial reflections arising from sample heterogeneity. Hence, from the FD data alone we can only with certainty assign the 0.47 nm distance as one of the dimensions of the unit cell. More specifically, this is the axis parallel to the fibril long axis (the orientation of which is known from the sample alignment), *i.e.* the classic β-strand spacing.

However, from the SAXS data we have an accurate assignment of one dimension, this being the 4.8 nm between consecutive protofilaments. When including this information, it is possible to assign the third dimension from the FD data. The unit-cell parameters are defined as *a* = 4.85, *b* = 3.21, *c* = 0.47 nm, α = β = γ = 90°. This indexing thus accounts for the position of the diffraction signals. The intensities of individual diffraction signals are dependent on the peptide packing in the unit cell and thus demand further consideration.

The unit-cell dimensions immediately suggest how many peptides are included in the repeating unit. We know that there is one layer of peptides in the unit cell, since the *c* axis is 0.47 nm, *i.e.* one β-strand. The length of one such heptapeptide β-strand is approximately 2.5 nm, immediately suggesting that two such peptides may span the longest dimension of the unit cell. From an inspection of the crystal packing of peptides, we know that peptide sheets can pack with dimensions of 1.1–1.5 nm, *i.e.* two such sheets of peptides can pack along the *b* axis (3.21 nm). The immediate suggestion from the unit-cell dimensions is thus that four peptides pack in the unit cell.

To validate this assumption, we calculated the corresponding solvent content of the unit cell. The unit cell has a volume of 7.3 nm^3^, meaning that a solvent content of 45% and a Matthews coefficient of 2.2 Å^3^ Da^−1^ result from the positioning of four peptides in the unit cell. This value is within the normal expected range in crystal packing (Kantardjieff & Rupp, 2003 ▶). Although not directly comparable to the packing of peptides in fibrils, we do expect a near-crystalline protein density and a tight packing in the fibril structures (Sunde *et al.*, 1997 ▶).

A further validation of the number of peptides in the unit cell can be derived from the SAXS data. SAXS data from a highly elongated structure allow the determination of the mass per unit length (Feigin & Svergun, 1987 ▶). We have calculated this for the samples with high fibril content (see Supplementary Table S5 and §[Sec sec2]2 for details). The mean value for the 3.4–9.0 h samples with >99% fibril content is 41.6 ± 5.1 kDa nm^−1^, corresponding to 50 ± 6 peptide monomers per length unit (*i.e.* per nanometre; Supplementary Table S5). The β-strand spacing in the sheets is defined from FD as 0.47 nm, and these sheets are formed along the fibril long axis. Thus, the SAXS-based mass estimate corresponds to 24 ± 2 monomers in a single-layered cross-section of 0.47 nm thickness. The second unit-cell dimension (4.85 nm) is already assigned to the individual protofilament width, and thus corresponds to the highly repetitive distance observed in the SAXS data, thus resulting in a Bragg peak. Our SAXS-based dimension of the cross-section suggests that we have an elongated cross-section with a maximal dimension of approximately 40 nm. This would mean that a maximum of 40/4.85 = 8.2 unit cells could pack along the longest dimension of the cross-section, resulting in [(24 ± 2)/8 = 2.8–3.3 ≃ 3] three peptides per unit cell. The geometric fitting of the SAXS dimensions suggests a longest cross-section dimension of 35–40 nm, corresponding to 3–4 peptides in the unit cell, by following the same logic. TEM images reveal a significant variation in the ribbon width in the TEM images. The mass per unit length estimate from SAXS is the average of all solution species, while the maximum width of 40 nm represents the largest structures. This means that the average number of peptides per unit is systematically underestimated. In conclusion, the mass per unit length analysis from the SAXS data supports the packing of four peptides in each unit cell.

In conclusion, each unit cell contains four peptide monomers, and this unit cell thus represents the protofilament cross-section.

The two latest samples (10.7 and 13.1 h) show both larger cross-sections as well as a larger estimated mass per unit length (Supplementary Table S5). The shift of the maximum in the cross-section *P*(*r*) function (Fig. 1 ▶ *e*) indicates that these samples are thicker, and we conclude that there is a hierarchical layering of the ribbons in these samples.

### 3.5. Approaching a quasi-atomic resolution structure of the fibril   

Determination of the unit-cell dimensions and the number of peptides per unit cell, together with previously published data (Marshall *et al.*, 2010 ▶), enables the conception of a detailed model of the fibril packing.

**Figure 3:**
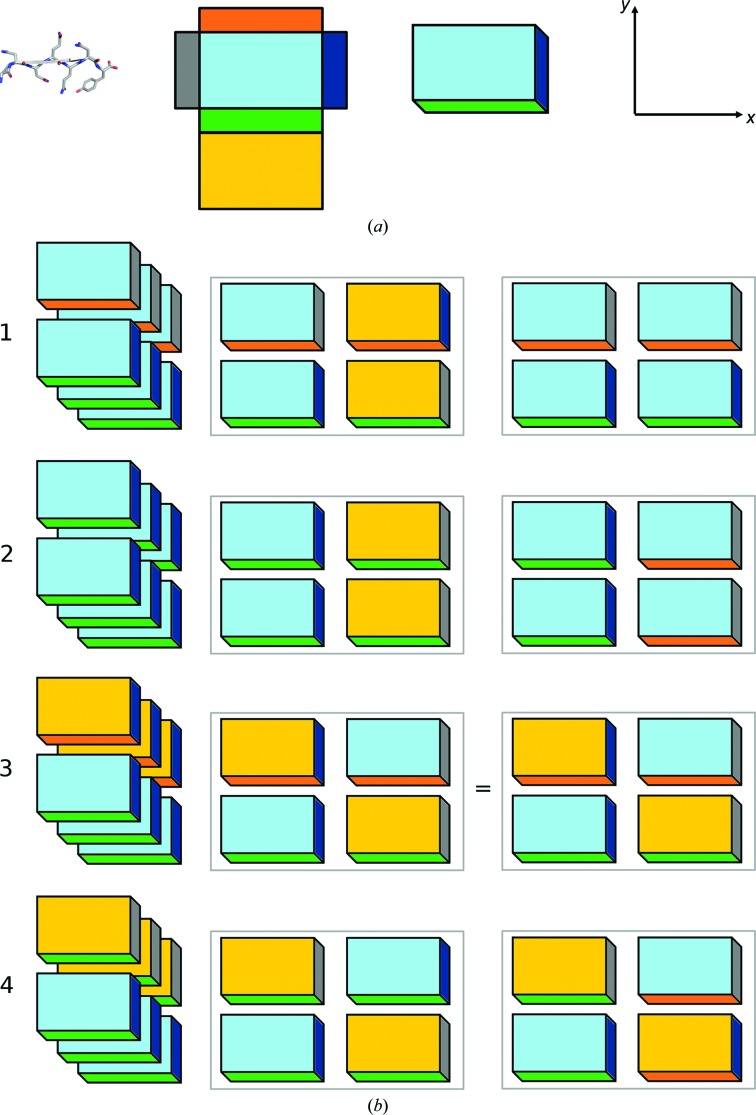
Packing of two-zipper motifs of class 1–4 zippers. (*a*) The zipper motif from the GNNQQNY crystal structure (PDB entry 2omm; Sawaya *et al.*, 2007 ▶) is shown along with a schematic representation of a single peptide by a box (inspired by the presentation in Stroud, 2013 ▶). The different colours differentiate between C/N-termini, odd/even-residue side chains and the up/down orientation of the given β-sheet. (*b*) Left column: single zipper motifs of classes 1 to 4 for which all individual sheets are parallel. Middle column: a second zipper motif generated by rotation around a twofold axis parallel to *y* (perpendicular to the fibril axis). Right column: a second zipper motif generated by rotation around a twofold axis parallel to *z* (corresponding to the fibril long axis or spine axis).

Zipper motifs formed by two tightly paired β-sheets have now been observed in numerous crystal structures of peptides (Nelson *et al.*, 2005 ▶; Sawaya *et al.*, 2007 ▶; Wiltzius *et al.*, 2008 ▶; Eisenberg & Jucker, 2012 ▶). Based on these structures, eight possible different sheet-to-sheet arrangements have been classified (Sawaya *et al.*, 2007 ▶; Eisenberg & Jucker, 2012 ▶) defined by their face-to-face interactions and parallel or antiparallel sheets. Furthermore, a recent rigorous derivation of the possible zipper groups revealed a total of ten different classes (Stroud, 2013 ▶). According to MD simulations, an asymmetric Gln/Asn-rich peptide such as GNNQQNY can form stable structures within all of the original eight classes (Berryman *et al.*, 2011 ▶). We observe a 0.47 nm meridional signal in our FD data, but not a signal at 2 × 0.47 nm in the meridional direction, which implies that the GNNQQNY β-sheets in these fibrils are parallel (Sikorski *et al.*, 2003 ▶) as in the crystal structures (Nelson *et al.*, 2005 ▶; Sawaya *et al.*, 2007 ▶), meaning that the possible packing motifs belong to one of four remaining classes (classes 1–4). These classes can be illustrated schematically, as shown in Fig. 3 ▶ (left column). Each β-strand has two distinct sides (with odd side chains projecting to one side and even side chains projecting to the other). If odd side chains pair with odd side chains in the neighbouring β-strand, this is a so-called face-to-face arrangement (classes 1 and 3 in Fig. 3 ▶; the orange side of the box is facing the orange side). Odd/even side-chain pairing is characteristic of classes 2 and 4 (the orange side faces the green side of the box in Fig. 3 ▶). These two β-strands are either arranged in parallel (classes 2 and 3; blue ends facing same way in Fig. 3 ▶) or antiparallel (classes 1 and 4; blue ends facing opposite ends and hence grey/blue pairs are visible in Fig. 3 ▶). We observe four peptides in our unit cell. These four peptides thus form two such zipper motifs, and the two consecutive zipper motifs can be organized in only two ways (by rotation either perpendicular or parallel to the fibril axis). This results in a total of eight arrangements, as depicted in Fig. 3 ▶ (note that the two arrangements within class 3 are identical). Note that combinations potentially formed by adding the second zipper motif by a simple translation of motif 1 are ruled out because this would lead to a unit-cell dimension of *a*/2, which we do not observe. The same criterion immediately rules out one class 1 arrangement (that on the right; obtained by rotation parallel to the fibril axis but yielding an arrangement corresponding to a pure translation) as a possible arrangement. When regarding the remaining six possible arrangements, classes 2 and 4 can also be ruled out as they have face-to-back arrangements (green facing orange in Fig. 3 ▶). This interaction could in principle be repeated indefinitely, meaning that it is illogical that only two peptides are zipped (using class 2 on the left in Fig. 3 ▶ as an example, a green face identical to the face that defines the interfacial contact in the zipper is exposed; thus, an orange face would easily access this green face). Thus, classes 2 and 4 have no actual boundaries in the *y* direction and are excluded as possible stacking principles yielding the observed unit cell. Likewise, class 1 (left) has no boundaries in the *x* direction. We can thus exclude both class 1 motifs (that on the left because of the lack of boundaries and that on the right because it defines a unit cell with an *x* axis of *a*/2) and we thus conclude that the peptide motif in the fibril is distinctly different from the peptide motif in the crystal structures, which are both class 1 zippers (Nelson *et al.*, 2005 ▶; Sawaya *et al.*, 2007 ▶). Two-zipper motifs from classes 1, 2 and 4 thus do not fit the observed data. This means that we can identify class 3 as the packing motif used in the formation of GNNQQNY fibrils.

**Figure 4:**
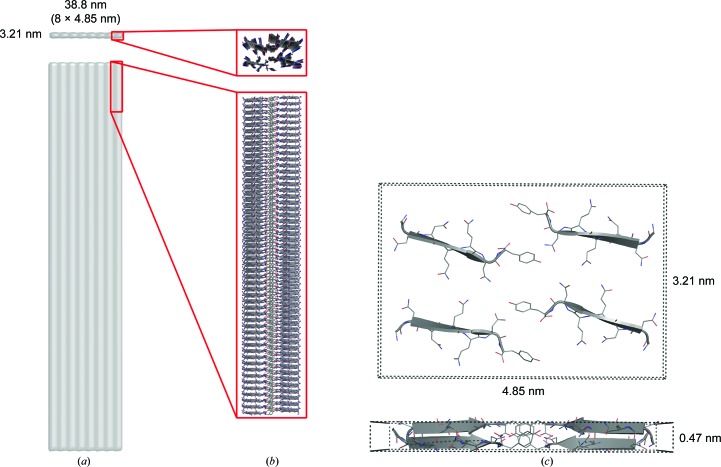
Schematics of the ribbon and the packing motif. (*a*) A flat ribbon, here depicted with eight protofilaments side by side. (*b*) Stacks of β-strands in the protofibril. (*c*) The corresponding unit cell with the suggested packing motif.

The final suggested packing motif therefore consists of two class 3 zippers. In the suggested motif the zippers are formed face to face, presumably with the even-numbered residues (Asn2, Gln4 and Asn6) forming the zipper (Fig. 4 ▶), resembling the internal sheet-to-sheet interaction observed in the crystals. The motif of the two zippers indicates interactions of either all four terminal glycines (Gly1) or four tyrosines (Tyr7). Importantly, tyrosine mobility has previously been noted as a distinction between GNNQQNY crystal forms and fibrils (van der Wel *et al.*, 2010 ▶) and in results from linear dichroism (LD), which indicates that the stacking of Tyr7 in the fibrils is perpendicular to the fibril long axis (Marshall *et al.*, 2010 ▶). Although the charged terminals are close in the suggested packing arrangement, the electrostatic forces appear to be less important for aggregation stability, which instead appears to be dominated by van der Waals interactions, thereby compensating for otherwise unfavourable electrostatic interactions (Berryman *et al.*, 2011 ▶). No peptide crystal structures with class 3 zipper motifs have been reported (Eisenberg & Jucker, 2012 ▶); however, GNNQQNY has also been shown to retain the ordered cross-β structure in this configuration in MD simulations (Berryman *et al.*, 2011 ▶). In addition, this suggested two-zipper motif shows distinct boundaries, which may well explain the difference between the fibrils and crystals. In the case of GNNQQNY, it may even be speculated that the initial interaction of two sheets [up–up or up–down, corresponding to the difference between class 1 (observed in crystals) and class 3 (suggested)] determines the pathway to either crystal or fibril.

Based on the suggested overall structural motif, and the possible tyrosine interactions, a model of the unit cell was constructed (Fig. 4 ▶). The corresponding fibre diffraction patterns were simulated using *CLEARER* (Makin *et al.*, 2007 ▶) and the major meridional and equatorial reflections are comparable to the experimental data (Fig. 2 ▶ *d*).

## 4. Discussion   

### 4.1. Peptide fibrillation may proceed through monomer addition and not *via* transient oligomers   

No buildup of oligomeric intermediates was observed during the GNNQQNY fibrillation process. Instead, the minor third component detected in this study is likely to be a result of changes in the maturation phase. This is in contrast to previous studies (Vestergaard *et al.*, 2007 ▶; Giehm *et al.*, 2011 ▶; Oliveira *et al.*, 2009 ▶) on other fibrillating systems, where volume fractions of up to 60% of transiently formed oligomers were observed. A possible explanation is that a given full-length protein requires a larger degree of refolding before assuming a fibrillation-prone conformation, and such a conformation may be stabilized in intermediate oligomers. The surface of such oligomers (or structural nuclei) may associate, potentially complemented by direct monomer addition (Vestergaard *et al.*, 2007 ▶; Oliveira *et al.*, 2009 ▶; Giehm *et al.*, 2011 ▶). The heptapeptide is in a monomeric native starting conformation that is expected to be rapidly fluctuating between different conformations, including an extended β-like conformation (Strodel *et al.*, 2007 ▶), some of which may be prone to fibrillation. Hence, the assembly of an intermediate oligomer may no longer be a prerequisite for fibril formation, even though one (or a few) of the conformations included in the ensemble of structures can still be considered to be the (thermodynamic and/or structural) nucleus. The structural equilibrium of the monomeric ensemble of structures is evidently dependent on experimental conditions, for example temperature and peptide concentration; hence, the rate of fibrillation is influenced by several factors. As an example, we keep the dissolved peptide at low temperatures to avoid the immediate onset of fibrillation. Under the conditions investigated, we detected only a brief lag phase followed by an exponential growth phase, which is typically assigned to the dependence on secondary nucleation. This could be assigned to fibril surface effects, where the fibril surface auto-catalyzes fibril elongation. In our framework, this would correspond to a shift in the structural equilibrium of the monomeric species, promoting a higher proportion of the fibrillation-prone conformation among the ensemble of multiple possible monomeric peptide conformations. We conclude that the lack of evidence for oligomeric forms of the heptapeptide does not exclude the possibility of a nucleation-dependent process or elongation by oligomer addition, but the nuclei and oligomers would then be formed in low numbers and exist within short time frames. Here, our observations do suggest that the elongation of GNNQQNY fibrils is dominated by monomer addition. Studies focused on a much larger so-called NM fragment (Sup35p_1–254_; Collins *et al.*, 2004 ▶) showed through fibrillation kinetics, analytical ultracentrifugation and single-molecule fluorescence that fibrillation proceeds *via* monomer addition. On the other hand, a study using a shorter N-terminal fragment (Sup35p_5–26_; Narayanan *et al.*, 2006 ▶) shows that small oligomers are critical for the fibrillation process. However, this latter study was performed in a dialysis setup following the diffusion of differently sized species, *i.e.* a significantly different setup. In addition, it is observed that a structural reorganization from an α-helical to a β-sheet structure only occurs upon incorporation into, or formation of, the fibrillar form. Thus, we speculate that this N-terminal oligomer, although potentially critical for fibrillation, is perhaps not on the pathway to the fibrils.

### 4.2. Protofilaments and ribbons are mostly straight rather than twisted and intertwined   

Our TEM images reveal striated, highly elongated, ribbons with only a few twists and turns, in accordance with previous data (Diaz-Avalos *et al.*, 2003 ▶; Marshall *et al.*, 2010 ▶; van der Wel *et al.*, 2007 ▶; Lewandowski *et al.*, 2011 ▶). Obviously, surface effects may influence the degree of ribbon twisting seen. TEM further clearly reveals that the protofilaments do not form bundles, as often observed for other fibrils (for example, as in Jiménez *et al.*, 2002 ▶; Vestergaard *et al.*, 2007 ▶). Fibrillar bundles presumably occur if several parts of each protofilament interact and/or if each individual protofilament twists. Based on the side-by-side ribbon association observed, the inter­action between protofilaments must have a clear directional preference. The protofilament itself must thus also be in a rather rigid, nontwisting macroscopic conformation to result in the observed linear arrangement. We thus conclude that individual protofilaments do not twist, which will also cause a low degree of twisting in the ribbon, in accordance with our observations. Our SAXS data are devoid of surface effects and also do not reveal the presence of significant regular twisting within the range of distances probed by the data. In agreement with this observation, the fibril samples are highly viscous, and we have observed a tendency for preferential orientation in the SAXS sample cell (evident as non-isotropic scattering signals when fibril samples were not sonicated; such data were discarded prior to analysis).

A previously published TEM analysis (Lewandowski *et al.*, 2011 ▶) reported striation widths of 5.1 ± 0.7, 7.1 ± 1.1 and 12.2 ± 1.2 nm. Bragg peaks are rather unusual in biomacromolecular SAXS, yet here one is clearly present corresponding to 4.8 nm arising from extremely well defined interpositioning of protofilaments, thus revealing the highly ordered macroscopic hierarchical buildup of these peptide fibrils.

A GNNQQNY fibre-to-crystal conversion has previously been reported (Marshall *et al.*, 2010 ▶). This was not detected in this work, where crystals were only rarely obtained after long-term storage at room temperature. Interestingly, the diffraction from these crystals does not match the simulated patterns based on the two known crystal structures (Supplementary Fig. S9), further underlining the polymorphism observed for this and several similar short peptides (Sawaya *et al.*, 2007 ▶; Berryman *et al.*, 2009 ▶).

### 4.3. A cross-disciplinary derived model of the atomic structure of GNNQQNY fibrils   

As described, the combination of the Bragg peak-containing SAXS data and the high-quality FD data enabled us to index the FD data. The SAXS-derived estimate of the mass per unit length further enables it to be concluded that there are four peptides within a unit cell of 4.85 × 3.21 × 0.47 nm. Finally, our cross-sectional analysis reveals that either one or two layers of protofilament ribbons form the final fibrils. Based on the available high-resolution crystal structures of amyloid-like peptide fragments, and a consideration of the unit-cell dimensions and protofilament packing, we further conclude that the GNNQQNY fibrils are packed as zippers of the class 3 type. We can thus provide a quasi-atomic resolution structure of the four-peptide unit cell, which utilizes available crystal structure peptide packing, modified also by the LD-based observation of a new orientation of the terminal tyrosine residue.

Previously published solid-state NMR analyses of GNNQQNY fibrils (Lewandowski *et al.*, 2011 ▶; van der Wel *et al.*, 2010 ▶) indicated the presence of three different backbone conformations (composite fibrils), and thus microscopically heterogeneous samples. We do not have a similar observation of microscopic heterogeneity in our samples, since the Bragg peak in the SAXS data is distinctly at one distance and we are able to fit our fibre diffraction data using one structure only. However, we have clear indications of macroscopic heterogeneity. As described in detail above, based on our SAXS data we observe the presence of ribbons of both one and two protofilaments in thickness, and we have clear evidence in our TEM data for the varying width of the ribbons. Finally, weak but random twisting of the ribbons is observed, further adding to the heterogeneous nature of the solution SAXS data. We can thus conclude that our samples are macroscopically rather heterogeneous, although microscopically apparently homogenous. The overall average parameters and dimensions can be obtained from the SAXS data as described above. It is not possible to fit our model to the SAXS data by including only one single model. The sample heterogeneity, which is clearly identified by both our cross-sectional analysis of SAXS data and the TEM data, means that in order to fit the SAXS data we would need to use a mixture of models, including ribbons of single/double protofilament thickness, varying large-scale twisting and possibly variations in ribbon width. Such fitting clearly includes too many parameters to be conclusive.

The detailed structure of amyloid fibrils from full-length proteins is still elusive. Usually, such detailed structural characterization of fibrils and the fibrillation process is hindered by the nature of fibrillation (a dynamic process including species on very different size scales), but when combining many methods, each with their own strengths, it is possible to overcome these limitations, in particular when studying shorter fragments of the amyloidogenic proteins. This has recently been demonstrated in a highly elaborate study of a transthyretin fragment (Fitzpatrick *et al.*, 2013 ▶) centred around numerous MAS NMR studies in combination with scanning transmission EM or cryo-EM and FD, providing a high-resolution final model, including a qualitative description of the heterogeneity of mature fibrils. Here, we have established a plausible atomistic model of the peptide structure in a highly hierarchical fibril packing. Based on more easily obtainable SAXS data, we qualitatively describe the fibril heterogeneity, and based on the same SAXS data we assign the FD unit cell. The method is hence significantly less laborious than using MAS NMR, but also results in a lower resolution model that does not provide details of side-chain positions but does reveal the overall packing scheme with clear boundaries with zipper structures in a so-called class 3 motif that has not previously been observed. The SAXS analysis in addition provides information about the fibrillation process, including potential analysis of additional species in the process. With an interest in protein fibrillation originating from disease or possible application as scaffolds for biocompatible materials, these approaches to the structural investigation are equally applicable. Investigating the fibrillation process and the fibrillar samples, we have demonstrated a hybrid method for fibril structure analysis which could in future also be extended to include models of full-length amyloid-like systems as well as other self-assembling systems.

## Supplementary Material

Additional figures and tables with details of the data analysis.. DOI: 10.1107/S1399004715001674/wa5080sup1.pdf
