# Supplementary material for: The architecture of amyloid-like peptide fibrils revealed by X-ray scattering, diffraction and electron microscopy
Source: Acta Crystallogr D Biol Crystallogr. 2015 Mar 27;71(Pt 4):882–95. doi: 10.1107/S1399004715001674 (PMC4388266; doi:10.1107/S1399004715001674)
Supplement: Supplementary file 1 [file d-71-00882-sup1.pdf]

BIOLOGICAL
CRYSTALLOGRAPHY

Volume 71 (2015)

Supporting information for article:

The architecture of amyloid-like peptide fibrils revealed by X-ray scattering, diffraction and electron microscopy

Annette E. Langkilde, Kyle L. Morris, Louise C. Serpell, Dmitri I. Svergun and Bente Vestergaard

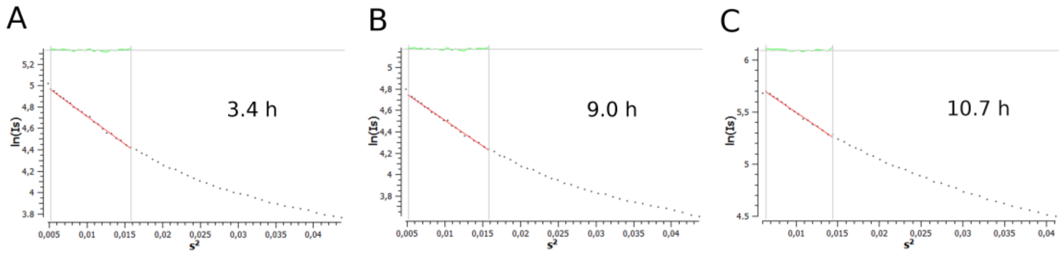

Figure S1. Guinier plots assuming rod-like particles. (A) 3.4 h, (B) 9.0 h and (C) 10.7 h samples, respectively, for which the corresponding cross section pair distance distribution functions are all shown in Figure 1E. Black dots are experimental data points, red lines are the Guinier fits and residuals are shown in green. Via the Guinier approximation the intercept $\lim_{s \to 0}[sI(s)]$ is obtained, which is then used to determine the mass-per-unit length as described in Materials and Methods.

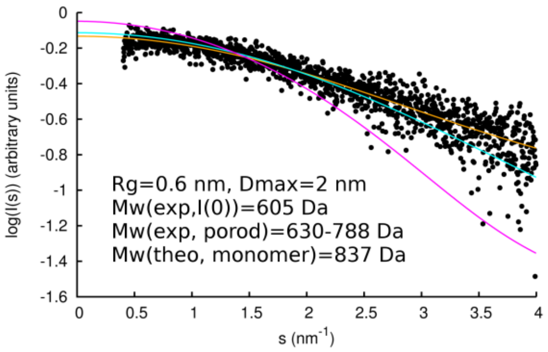

Figure S2. Scattering from the monomeric peptide. First SAXS data curve from the fibrillation series with 10% DMSO (0.2 h) compared to theoretical scattering curves. Experimental data (black), theoretical curves calculated from a monomeric peptide (–, χ = 0.523) and two different dimers: two neighboring peptides in a β-sheet (–, χ = 0.607) and the dry zipper β-strand pair (–, χ = 1.289).

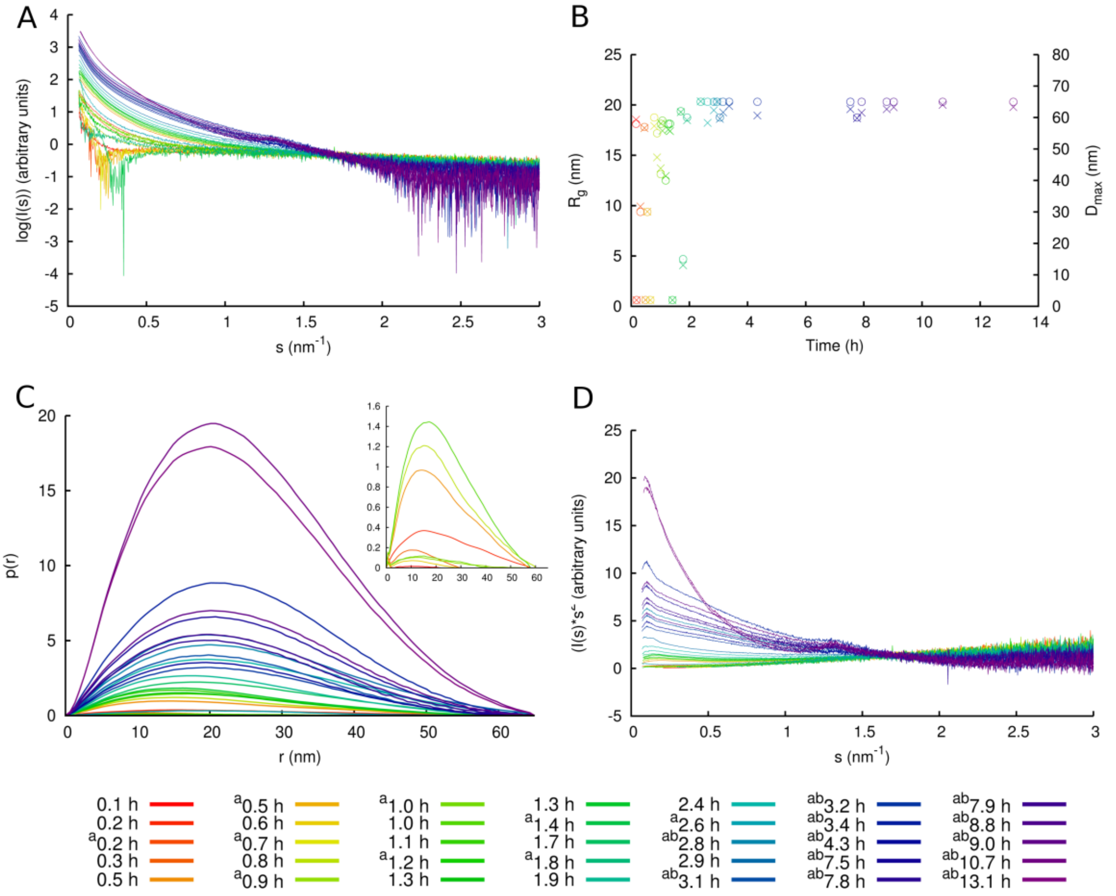

Figure S3. Additional SAXS data analysis. (A) SAXS data as Figure 2A, including all low angle scattering data points, i.e. also beyond the Guinier range. (B) R_g (crosses) and D_{max} (circles) of the extracted samples. (C) Pair distance distribution functions from the indirect Fourier transformation, insert highlights the development within the first hour. (D) Kratky plot of the SAXS data. The development over time is illustrated using a color scale from red to purple. [a]Fibrillation conditions include 10% DMSO. [b]The sample was sonicated immediately before measuring SAXS data.

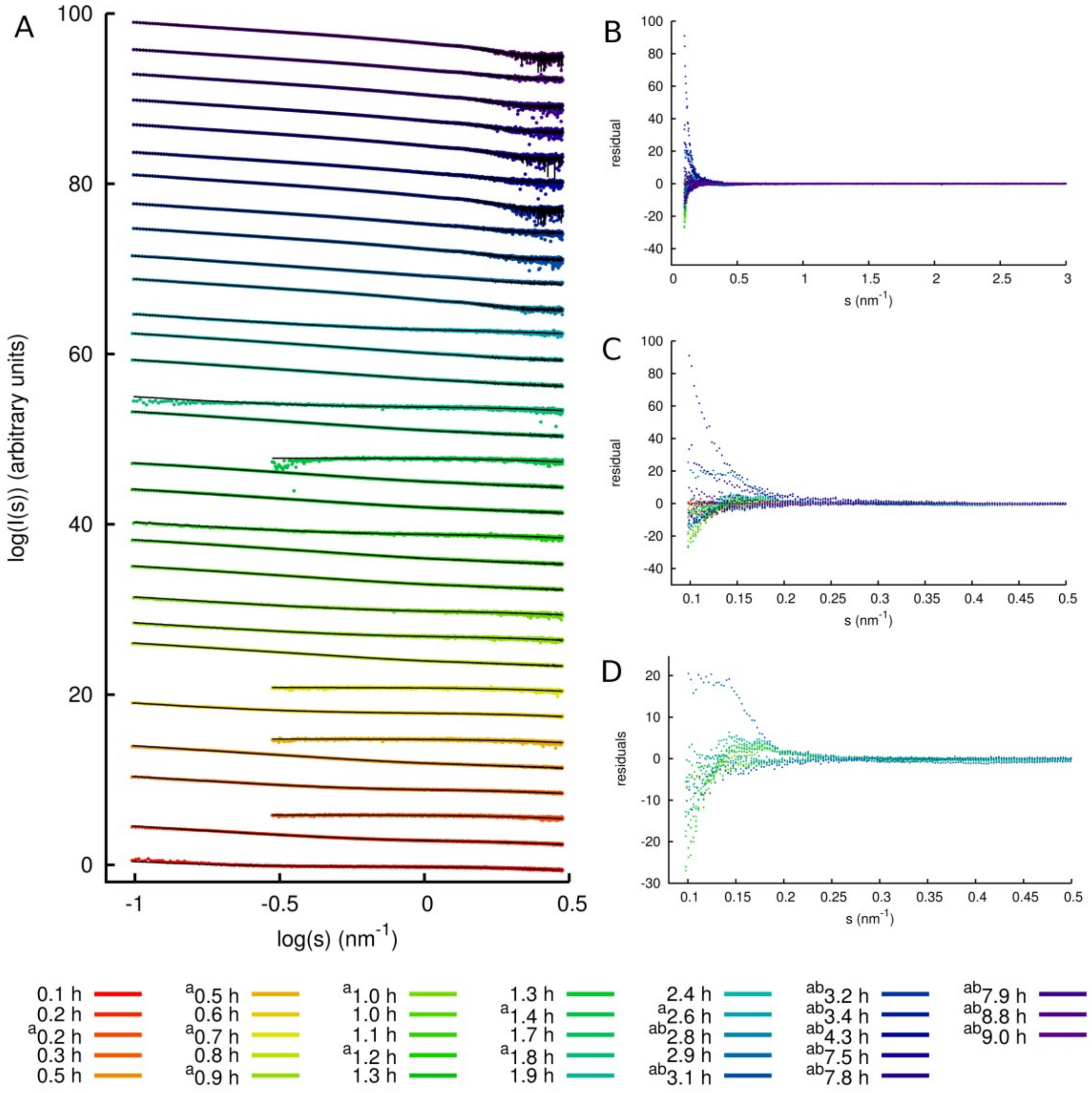

Figure S4. Fitting two main components to the SAXS data. Decomposition of the data series, excluding the 10.7 h and 13.1 h samples. Experimental data was fitted by linear combinations of monomer and fibril scattering, represented by a theoretical monomer and the 9.0 h sample respectively, using the program OLIGOMER. (A) Log-log plots of experimental data (colored according to the key) and corresponding calculated fits (black). Data from individual time-points are translated for viewing purposes. For four samples with monomer-like scattering only, the data is limited to >0.3 nm^{-1}. The corresponding volume fractions and fitting parameters are listed in Table S2 (B) Residuals from all fits. (C) Residuals from all fits, zoomed to low s range. (D) Residuals for the fit to samples in the 1.0 – 3.1 h time frame.

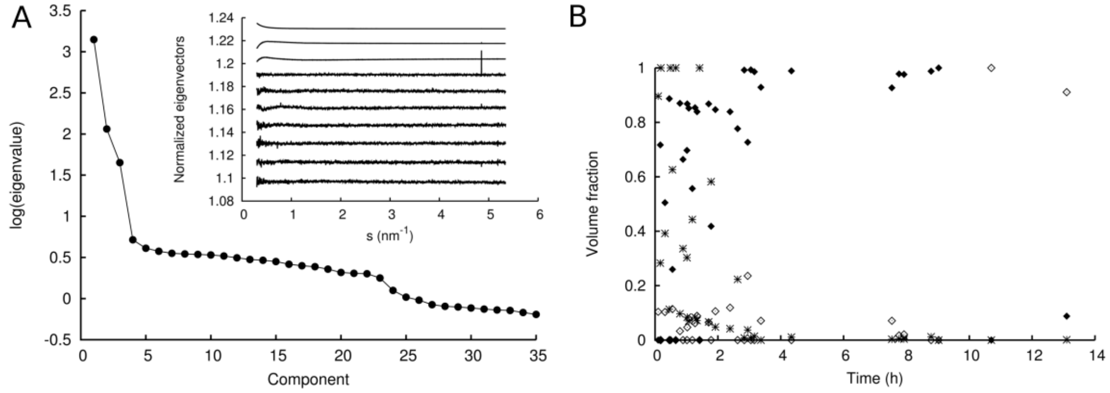

Figure S5. Analysing a possible third component. (A) SVD of all data curves, as supplement to Figure 1C (i.e. here including data also from 10.7 h and 13.1 h). Eigenvalues from the analysis and as insert, the first 10 eigenvectors are shown. (B) Volume fractions from a three component fit (cf. Fig. S6). Monomers (stars), represented by the theoretical scattering; and two different late states, represented by 9.0 h (filled diamonds) and 10.7 h (open diamonds), respectively.

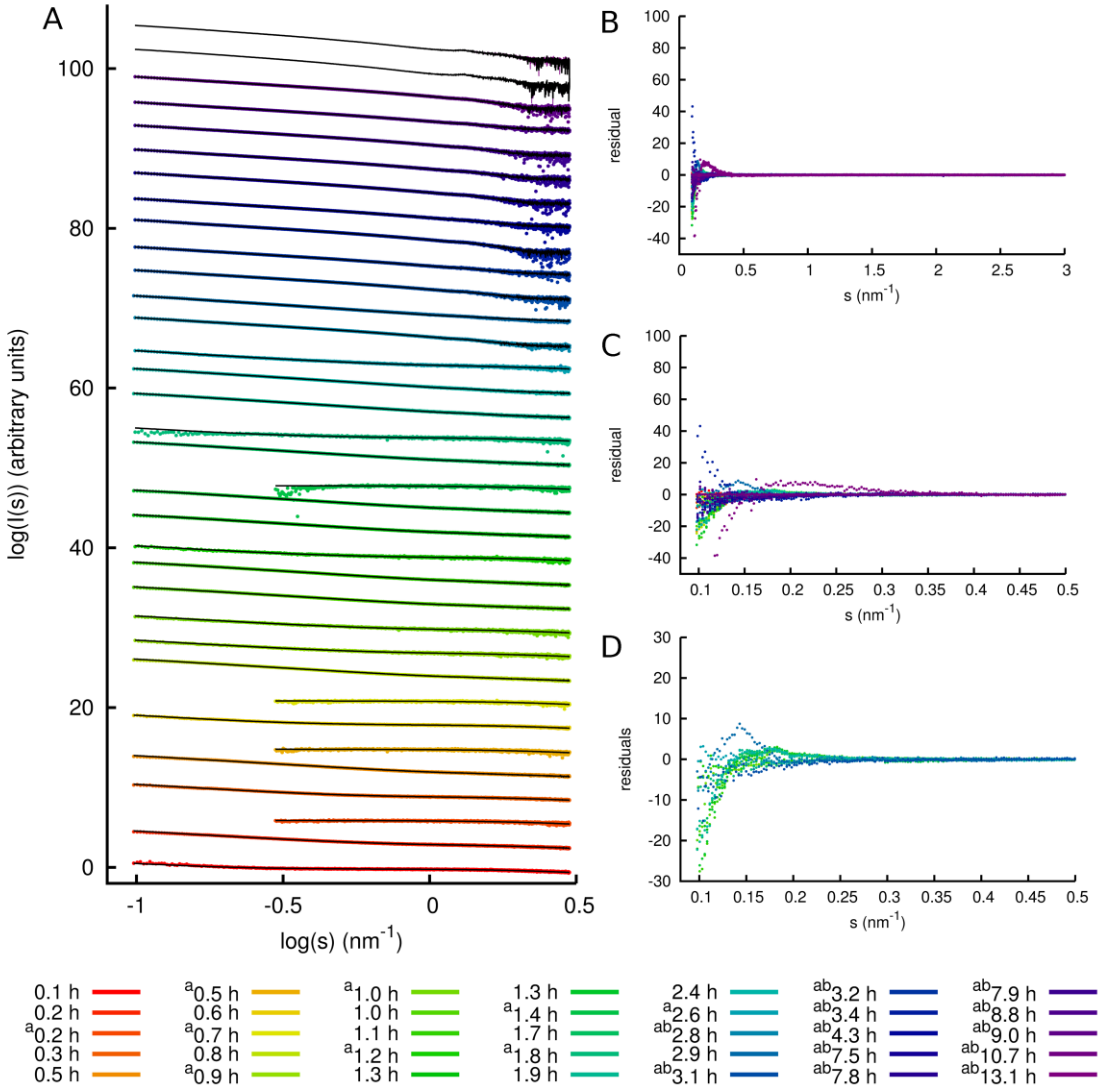

Figure S6. Three component fitting to the SAXS data. Decomposition of the entire data series using the program OLIGOMER. Experimental data was fitted by linear combinations of monomer and fibril scattering, represented by a theoretical monomer and both 9.0 h and 10.7 h samples, respectively. (A) Log-log plots of experimental data (colored according to the key) and corresponding calculated fits (black). Data from individual time-points are translated for viewing purposes. For four samples with monomer-like scattering only, the data is limited to >0.3 nm^{-1}. All calculated fits are black. The corresponding volume fractions are plotted in Fig. S5B. (B) Residuals from all fits. (C) Residuals from all fits, zoomed to low s range. (D) Residuals for the fit to samples in the 1.0 – 3.1 h time frame.

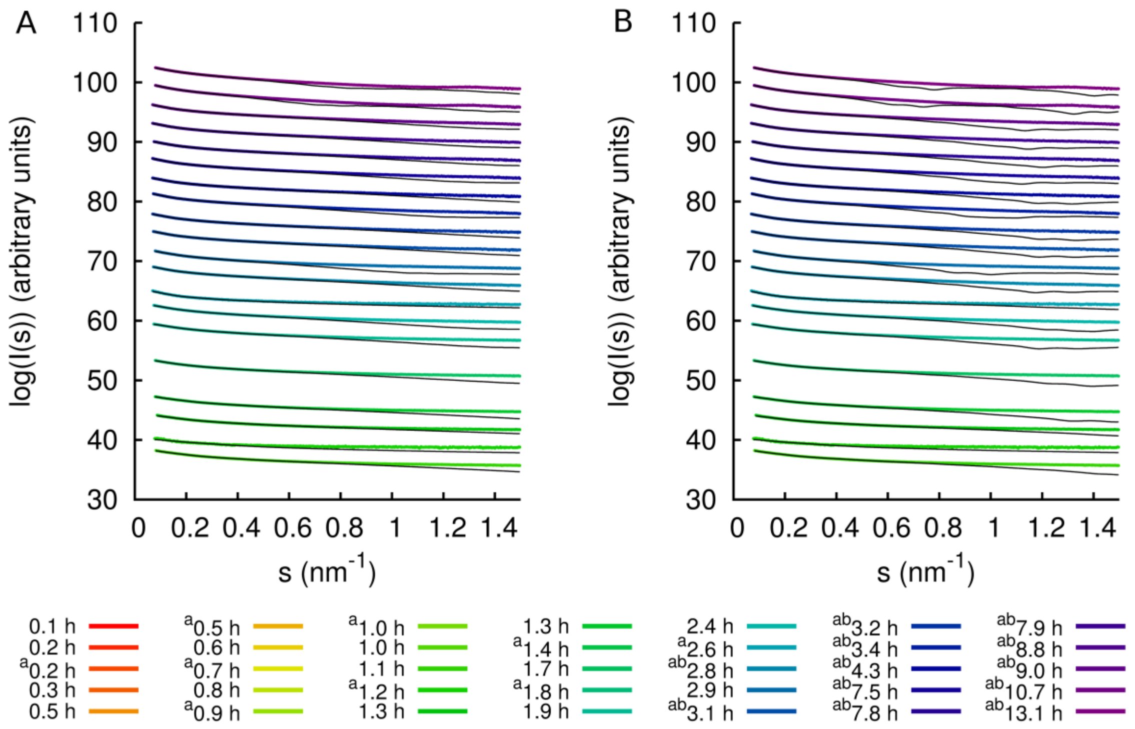

Figure S7. Fitting geometrical shapes to SAXS data. A cylindrical ellipsoid (A) or parallelepiped (B) was fitted to the low resolution SAXS data ($s < 1.5\ nm^{-1}$). Data from the earliest time points (less than 1.0 h) are excluded, as are the 1.4 and 1.8 h samples showing mainly monomer characteristics. All calculated curves are black, and the corresponding shape parameters and χ^2 values are listed in Table S3. Experimental data is colored according to the key.

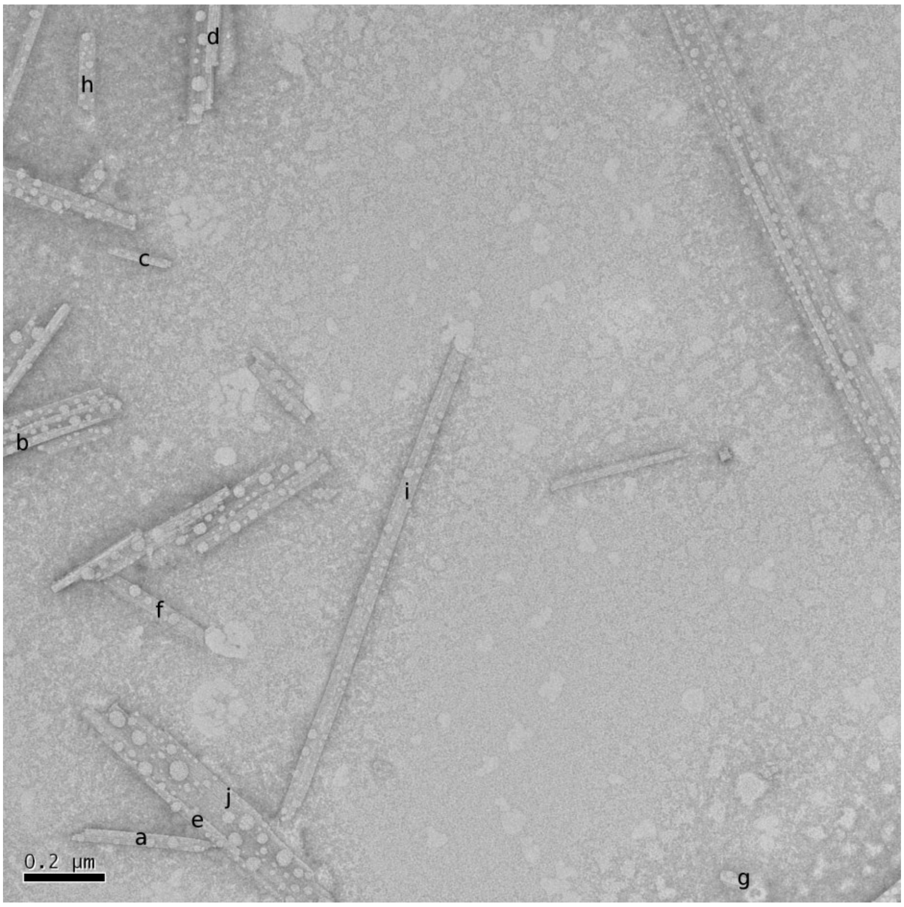

Figure S8. Determination of striation width from TEM. Enlarged version of Fig 2B showing sonicated fibrils with annotation (a-j) indicating ribbons measured for determination of striation width, cf. Table S4.

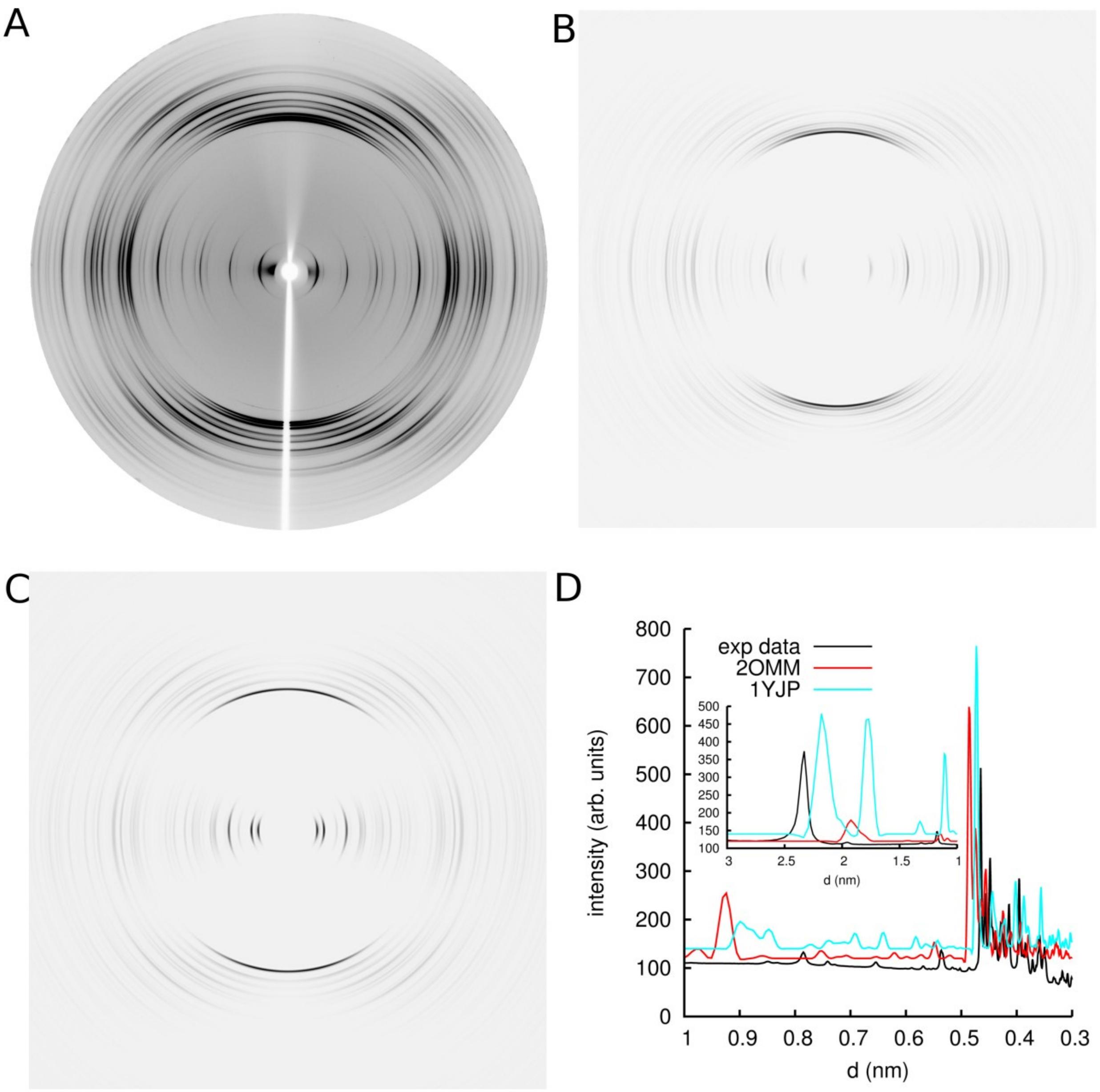

Figure S9. Comparison of crystal structures based on diffraction patterns. Diffraction from a GNNQQNY crystalline sample obtained during this study (A) and simulated patterns from known crystal structures (PDB entry 2OMM (B) and 1YJP (C)), respectively. (D) Corresponding 360° radial average of the three patterns.

Table S1. Indexing of signals in the fiber diffraction pattern.

Observed reflection (nm)	Indexing	Calculated reflection (nm)
4.8[a]	[1 0 0]	4.85
3.2[b]	[0 1 0]	3.21
1.6	[0 2 0]	1.61
1.35	[2 2 0]	1.34
0.93	[5 1 0]	0.93
0.81	[6 0 0]	0.81
0.76	[2 4 0]	0.76
0.70	[7 0 0]	0.69
0.60	[8 0 0]	0.61
0.51	[9 2 0]	0.51
0.46	[10 2 0]	0.46
0.40	[1 8 0]	0.40

[a]From SAXS Bragg peak [b]From the previously published pattern (Marshall, K.E. *et al. Biophys. J.* **98**, 330-8 (2010)).

Table S2. Volume fractions and χ^2 from the two component analysis.

Sample	χ^{2a}	Monomer fraction[b]	Fibril fraction[c]
0.1 h	1.29	0.875 ±0.002	0.125 ±0.003
0.2 h	2.06	0.277 ±0.000	0.723 ±0.001
0.2 h	0.52	1.000 ±0.006	0.000 ±0.000
0.3 h	1.67	0.370 ±0.001	0.630 ±0.001
0.5 h	4.93	0.110 ±0.000	0.890 ±0.000
0.5 h	0.54	1.000 ±0.009	0.000 ±0.000
0.6 h	1.16	0.598 ±0.001	0.402 ±0.002
0.7 h	0.56	1.000 ±0.008	0.000 ±0.000
0.8 h	4.70	0.091 ±0.000	0.909 ±0.000
0.9 h	0.54	0.329 ±0.003	0.671 ±0.008
1.0 h	0.60	0.297 ±0.003	0.703 ±0.008
1.0 h	3.94	0.078 ±0.000	0.922 ±0.000
1.1 h	5.70	0.062 ±0.000	0.938 ±0.000
1.2 h	0.57	0.436 ±0.004	0.564 ±0.010
1.3 h	4.09	0.076 ±0.000	0.924 ±0.000
1.3 h	5.44	0.063 ±0.000	0.937 ±0.000
1.4 h	0.75	1.000 ±0.009	0.000 ±0.000
1.7 h	3.71	0.060 ±0.000	0.940 ±0.000
1.8 h	0.60	0.574 ±0.006	0.426 ±0.015
1.9 h	4.70	0.039 ±0.000	0.961 ±0.000
2.4 h	4.83	0.033 ±0.000	0.967 ±0.000
2.6 h	0.51	0.217 ±0.002	0.783 ±0.006
2.8 h	0.55	0.008 ±0.000	0.992 ±0.001
2.9 h	9.89	0.022 ±0.000	0.978 ±0.000
3.1 h	0.56	0.007 ±0.000	0.993 ±0.001
3.2 h	0.54	0.014 ±0.000	0.986 ±0.002
3.4 h	0.95	0.000 ±0.000	1.000 ±0.001
4.3 h	0.60	0.010 ±0.000	0.990 ±0.002
7.5 h	0.84	0.001 ±0.000	0.999 ±0.001
7.8 h	0.66	0.005 ±0.000	0.995 ±0.001
7.9 h	0.63	0.003 ±0.000	0.997 ±0.001
8.8 h	0.54	0.011 ±0.000	0.989 ±0.001
9.0 h	0.01	0.000 ±0.000	1.000 ±0.001

[a]The $\chi 2$ values are dependent on the error estimation of the collected SAXS data. The data were collected during two different beamtime allocations. [b]Monomer represented by the theoretical monomer (Fig S1). [c]Fibril represented by the experimental data from the 9.0 h sample.

Table S3. Parameters from the fitting of geometrical shapes to the SAXS data.

Sample	Elliptical cylinder				Parallelepiped			
	a[a]	b[a]	c	χ[b]	a	b	c	χ[b]
13.1 h	4.3	18.6	46.6	1.934	8.8	32.8	49.5	1.012
10.7 h	4.8	21.1	55.2	0.819	9.5	34.4	52.2	0.911
9.0 h	2.5	21.5	52.7	0.800	5.7	34.3	50.4	1.179
8.8 h	2.3	21.5	52.0	0.629	5.3	34.0	49.6	0.981
7.9 h	2.5	21.2	52.3	0.677	5.7	33.9	49.8	1.030
7.8 h	2.4	20.3	49.3	0.575	5.5	32.4	47.2	0.879
7.5 h	2.7	21.7	52.9	0.870	6.0	34.6	50.6	1.208
4.3 h	2.2	20.9	51.2	0.474	5.2	33.2	48.8	0.815
3.4 h	2.9	21.9	53.4	1.041	6.4	35.2	50.8	1.285
3.2 h	2.2	20.8	50.8	0.485	5.1	32.9	48.5	0.820
3.1 h	2.3	20.9	50.8	0.517	5.3	33.1	48.3	0.854
2.9 h	3.3	19.2	48.2	1.029	6.8	31.2	45.4	1.180
2.8 h	2.4	21.0	51.1	0.582	5.4	33.3	48.7	0.918
2.6 h	0.6	31.8	37.2	0.566	1.1	36.0	54.4	0.722
2.4 h	2.6	18.4	47.8	0.812	4.9	27.5	62.9	1.263
1.9 h	2.5	17.1	45.5	0.575	5.2	27.7	43.3	0.803
1.8 h	2.2	16.8	44.5	0.564	4.7	27.1	42.1	0.775
1.3 h	2.0	14.9	55.5	0.285	4.6	25.2	38.9	0.600
1.3 h	1.5	14.3	37.4	0.715	3.3	22.8	36.1	0.938
1.2 h	0.3	7.9	38.4	0.621	0.6	13.5	38.4	0.626
1.1 h	1.9	14.4	54.8	0.283	4.0	23.2	54.2	0.454

[a]Semi-axis a and b describe the elliptical cross-section, i.e. these values should be doubled for comparison with the parameters for the parallelepiped. [b]χ is given for the fit between the experimental data and the theoretical scattering from the given shape for $s<1.5\ nm^{-1}$.

Table S4. Striation width from TEM.

Ribbon (cf. Fig. S8)	Measured width (nm)	No. of filaments	Filament width (nm)
a	33.9	7	4.85
b	22.5	4	5.62
c	24.9	5	4.97
d	32.0	6	5.33
e	30.5	6	5.09
f	36.3	7	5.18
g	23.2	5	4.64
h	39.9	8	4.98
i	44.0	9	4.89
j	34.2	7	4.88
			5.04±0.26

Table S5. Mass-per-length (M_L) estimates.

Sample	Mass-per-length (kDa nm^{-1})	No. of monomers[a] per nm	Fibril volume fraction[b]
3.1 h	22.6	27	0.993
3.2 h	17.7	21	0.986
**3,4 h**[c]	**48.9**	**58**	**1.000**
4,3 h	19.7	24	0.990
**7,5 h**[c]	**37.5**	**45**	**0.999**
7,8 h	27.9	33	0.995
7,9 h	30.5	36	0.997
8,8 h	23.9	29	0.989
**9,0 h**[c]	**38.5**	**46**	**1.000**
10.7 h	122	146	-
13.1 h	112	134	-

[a]Using a theoretical weight of 0.837 kDa for the monomeric peptide. [b]From the oligomer analysis (Table S2). [c]The three samples with > 99 % fibrils, mean M_L 41.6±5.1 kDa nm^{-1}, corresponding to 50±6 monomers per nm.
